# Evaluation of Secondary Concentration Methods for Poliovirus Detection in Wastewater

**DOI:** 10.1007/s12560-018-09364-y

**Published:** 2019-01-05

**Authors:** Jill C. Falman, Christine S. Fagnant-Sperati, Alexandra L. Kossik, David S. Boyle, John Scott Meschke

**Affiliations:** 1 Department of Environmental & Occupational Health Sciences, University of Washington, 4225 Roosevelt Way NE, Suite 100, Seattle, WA 98195 USA; 2 PATH, 2201 Westlake Avenue, Suite 200, Seattle, WA 98121 USA

**Keywords:** Environmental surveillance, Skimmed-milk flocculation, Wastewater, Enteric viruses, Poliovirus

## Abstract

Effective surveillance of human enteric viruses is critical to estimate disease prevalence within a community and can be a vital supplement to clinical surveillance. This study sought to evaluate simple, effective, and inexpensive secondary concentration methods for use with ViroCap™ filter eluate for environmental surveillance of poliovirus. Wastewater was primary concentrated using cartridge ViroCap filters, seeded with poliovirus type 1 (PV1), and then concentrated using five secondary concentration methods (beef extract-Celite, ViroCap flat disc filter, InnovaPrep® Concentrating Pipette, polyethylene glycol [PEG]/sodium chloride [NaCl] precipitation, and skimmed-milk flocculation). PV1 was enumerated in secondary concentrates by plaque assay on BGMK cells. Of the five tested methods, PEG/NaCl precipitation and skimmed-milk flocculation resulted in the highest PV1 recoveries. Optimization of the skimmed-milk flocculation method resulted in a greater PV1 recovery (106 ± 24.8%) when compared to PEG/NaCl precipitation (59.5 ± 19.4%) (*p* = 0.004, t-test). The high PV1 recovery, short processing time, low reagent cost, no required refrigeration, and requirement for only standard laboratory equipment suggest that the skimmed-milk flocculation method would be a good candidate to be field-validated for secondary concentration of environmental ViroCap filter samples containing poliovirus.

**Electronic supplementary material:**

The online version of this article (10.1007/s12560-018-09364-y) contains supplementary material, which is available to authorized users.

## Introduction

Effective surveillance of human enteric viruses is critical to estimate disease prevalence within a community, as enteric viruses are responsible for the majority of acute waterborne diseases worldwide (Wyn-Jones and Sellwood 2001; Fong and Lipp 2005; Maunula et al. 2009; Sinclair et al. 2009). Clinical surveillance is limited to infected, symptomatic individuals with access to and who seek health care (Mangal et al. 2013; Manor et al. 2014; Asghar et al. 2014). Enteric viruses primarily infect and multiply within the gastrointestinal tract of their host and are shed at high concentrations in fecal matter (typically between 10^5^ and 10^11^ virions per g stool) for up to several months (Salim et al. 1990; Blacklow and Greenberg 1991). Thus, they can be detected in wastewater treatment plant influents and effluents as well as surface waters in areas with poor sanitation (Petrinca et al. 2009; Gibson et al. 2011; Masachessi et al. 2017). Therefore, environmental surveillance can be a vital supplement to clinical surveillance by testing wastewater and wastewater-impacted surface waters for the presence of viruses, thereby capturing information on a community’s health (Hovi et al. 2012; World Health Organization [WHO] 2015a).

Environmental surveillance of enteric viruses includes sample collection and primary and/or secondary concentration of environmental waters, followed by analysis targeting the pathogens of interest. Primary concentration methods used today for concentration of enteric viruses in water typically involve electropositive or electronegative filtration media to process large sample volumes (up to 1000 L drinking water or groundwater) (Abbaszadegan et al. 1993; Ikner et al. 2012). Early methods for enteric virus surveillance were developed in the 1960s and 1970s and included aqueous polymer two-phase separation (hereafter, two-phase separation), hydroextraction, soluble ultrafiltration, and ultracentrifugation (Cliver and Yeatman 1965; Shuval et al. 1967, 1969; Grabow 1968; Rao and Labzoffsky 1969; Nupen 1970; Hill et al. 1971). These early methods were limited because they generally processed small sample volumes (~ 0.5–1 L) (Ikner et al. 2012) and are not considered feasible for large volume processing. More recently, they have been used as secondary concentration methods for improved viral detection following primary concentration by other methods.

Additional secondary concentration methods for enteric viruses include polyethylene glycol (PEG)/sodium chloride (NaCl) precipitation, organic flocculation, aluminum hydroxide precipitation-hydroextraction, and adsorption-elution (Ikner et al. 2012). Many previous studies have examined secondary concentration of enteric viruses from tap water and other relatively clean water sources (Wallis et al. 1970; Hill et al. 1972, 1976; Katzenelson et al. 1976; Farrah et al. 1977; Sobsey and Jones 1979; Sobsey et al. 1981; Dahling and Wright 1986a; Ma et al. 1994; Wen Li et al. 1998; Karim et al. 2009; Ikner et al. 2011; Rhodes et al. 2011). However, enteric viruses are most prevalent in wastewater, which is characterized by high organic matter and turbidity. Therefore, applicable concentration methods should be optimized to maximize the effective volume assayed while also considering these organic and inorganic inhibitors that could affect viral recovery efficiency (Ijzerman et al. 1997; Gibson et al. 2012; Schrader et al. 2012).

The most comprehensive environmental surveillance program is for poliovirus. Poliovirus is the only human enteric virus close to eradication, and water has been implicated in its transmission (Leclerc et al. 2002; Lodder et al. 2012). In 2017, there were only 22 wild poliovirus and 96 vaccine-derived poliovirus cases globally (WHO 2018a, b). Since the majority of those infected are asymptomatic, environmental surveillance can provide valuable data on viral circulation in the absence of clinical cases (Hovi et al. 1986; Minor 2016). Therefore, routine surveillance of wastewater and wastewater-impacted surface waters supplements disease-based clinical surveillance of acute flaccid paralysis for monitoring poliovirus transmission in communities. Environmental surveillance continues to expand as a tool to help identify poliovirus in endemic areas, warn of new importations, and certify elimination of vaccine strains (WHO 2013). Similarly, there is an expectation that environmental surveillance activities will expand for other vaccine-preventable diseases such as gastrointestinal diseases from *Salmonella enterica* serotype Typhi, rotavirus, norovirus (vaccine undergoing evaluation in clinical trials), and hepatitis A virus (Aw and Gin 2010; Fumian et al. 2011; Hellmer et al. 2014; Yanez et al. 2014; Zhou et al. 2016; Kazama et al. 2017; Riddle et al. 2018).

The WHO Guidelines for Environmental Surveillance of Poliovirus provide recommendations for the concentration of poliovirus from wastewater. Grab sample collection followed by two-phase separation is the WHO’s recommended method for environmental surveillance (WHO 2015b). This method processes 500 mL from a 1-L grab sample. The widespread use of this method is attributed to the reasoning that wastewater contains high viral loads due to the large quantities of enteric viruses shed in fecal matter making a 500-mL sample adequate for positive detection. However, as poliovirus approaches eradication, fewer infectious viral particles are present in waste streams, and the overall sensitivity of detection is affected by the sample volume collected and concentration methods employed. The bag-mediated filtration system (BMFS) was developed to concentrate larger volumes (3–10 L) in the field and in low-resource settings (Fagnant et al. 2014, 2018). The BMFS uses the virus adsorption and elution (VIRADEL) method for primary concentration of poliovirus based on electrostatic interactions between electronegatively charged poliovirus and the positively charged ViroCap™ filter, and it uses PEG/NaCl precipitation method for secondary concentration. The BMFS method was compared to the recommended WHO two-phase separation method through a field-validation study in Pakistan for environmental surveillance of poliovirus in selected source waters and resulted in improved poliovirus detection (WHO 2015b; Zhou et al. 2018).

This study sought to evaluate simple, fast, and inexpensive secondary concentration methods to use with ViroCap filter eluate for environmental surveillance of poliovirus. Beef extract-Celite (Dahling and Wright 1986a; Melnick 1996; Rhodes et al. 2011), ViroCap flat disc filtration, concentration by InnovaPrep® Concentrating Pipette, PEG/NaCl precipitation, and skimmed-milk flocculation (Calgua et al. 2008) were adapted as secondary concentration methods and evaluated following primary concentration of wastewater using ViroCap filters. The Concentrating Pipette and skimmed-milk flocculation methods were optimized, as poliovirus recovery from complex water matrices has not been previously characterized by these relatively new methods.

## Materials and Methods

### Virus Preparation and Cell Culture

Stocks of poliovirus type 1 (PV1) vaccine strains were prepared by confluent lysis of buffalo green monkey kidney (BGMK) cell monolayers (Sobsey et al. 1978). Viruses were extracted with chloroform, and purified stocks were stored at − 80 °C. BGMK cells were grown in 75-cm^2^ flasks or 9.5-cm^2^ wells containing Eagle’s minimum essential media (Corning Inc, Corning, NY, USA) supplemented with 10% fetal bovine serum (American Type Culture Collection, Manassas, VA, USA) at 37 °C with 5% CO_2_.

### Water Samples

Ten-liter samples of influent wastewater were collected from a wastewater treatment plant in Seattle, WA. The water was stored at 4 °C, used within 24 h, and thoroughly mixed before use.

### Primary Concentration

Samples were prepared by passing 10-L influent wastewater through a positively charged 2″ ViroCap filter (Scientific Methods, Granger, IN, USA) using a peristaltic pump. ViroCap filters have an average pore size of 2–3 µm and contain glass microfibers coated with alumina nanofibers (Karim et al. 2009). After filtration, the filters were stored at 4 °C for up to 4 h and then eluted by adding 100-mL sterile eluent (1.5% beef extract [Becton, Dickinson and Company, Franklin Lakes, NJ, USA], 0.05 M glycine [TCI, Tokyo, Japan], pH 9.5) to the filter inlet. After a 30-min filter contact time, the eluate was recovered via the filter outlet. The recovered eluate (pH 8.5–9.5) was immediately pH-adjusted to 7.0-7.5 using 1 M HCl and, if the pH was over-adjusted, 1 M NaOH. The pH-adjusted filter eluate (100 mL) was stored at − 20 °C until seeding and secondary concentration and is referred to here as the primary concentrate.

### Secondary Concentration Preliminary Investigations

Preliminary investigations involved adaptation of each secondary concentration method for use with the primary concentrate from ViroCap filters. Secondary concentration methods tested in preliminary investigations included: (1) beef extract-Celite; (2) ViroCap flat disc filter; (3) Concentrating Pipette; (4) PEG/NaCl precipitation; and (5) skimmed-milk flocculation. The secondary concentration step was evaluated for PV1 recovery independently of PV1 recovery during primary concentration. During this evaluation, 10^3^ to 10^4^ PFU PV1 were spiked into 100-mL primary concentrate and mixed thoroughly by shaking (10–15 min, 200 RPM, room temperature [20–25 °C]). All samples were concentrated to a final volume of 10–20 mL, and pH adjustments were performed using 1 M HCl and 1 M NaOH.

#### Beef Extract-Celite

Secondary concentration of the primary concentrate by beef extract-Celite was performed as previously described (Rhodes et al. 2011). Briefly, 0.1 g Celite (Sigma-Aldrich Celite® 577, fine, St. Louis, MO, USA) was added per 100 mL of sample. Samples were shaken (10 min, 200 RPM, room temperature) and vacuum filtered using an AP20 filter, with a 2.0 µm pore size and glass fiber filter with binder resin (Millipore Corp, Bedford, MA, USA). Viruses were eluted from the Celite by passing 10–20 mL phosphate-buffered saline (PBS), pH 9.0 through the filter. This final PBS eluate was collected, pH-adjusted to 7.0–7.5, and assayed.

#### ViroCap Flat Disc Filter

For secondary concentration using a ViroCap flat disc filter, the spiked primary concentrate was diluted with an additional 500 mL of sterile deionized water and adjusted to pH 7.0–7.5. Samples were vacuum-filtered through a 60-mm diameter ViroCap flat disc filter (Scientific Methods), and the filtrate was collected and assayed to determine viral particle filter bypass. The ViroCap disc filter was then removed using sterile forceps and placed in 10-mL eluent (1.5% beef extract, 0.05 M glycine, pH 9.5) for a 30-min filter contact time. The ViroCap disc filter and eluent were then returned to the magnetic filter holder, and the eluent was vacuum filtered to elute viruses remaining on the filter media. The eluent was collected, pH-adjusted to 7.0–7.5, and assayed.

#### Concentrating Pipette

Concentration by the Concentrating Pipette (InnovaPrep, Drexel, MO, USA) was performed on the primary concentrate using ultrafiltration hollow fiber polysulfone pipette tips (65 kDa [25–30 nm cutoff] pore size, 98 cm^2^ surface area). Additional experiments evaluating different sample matrices, instrument settings, number of extractions, and extraction fluids were also performed (Online Resource 1). The 100-mL spiked matrix was divided into two 50-mL aliquots for concentration, as the pipette tips fouled after processing 50 mL. Custom instrument settings recommended by the manufacturer were used: valve open, 35; valve closed, 100; pulse count, 2; flow buffer, 12; and extraction delay, 6. Following concentration, one or two extractions were performed using 0.075% Tween 20 Tris (HC08001, InnovaPrep). Each extraction used approximately 1–1.5 mL of elution fluid. (Fluid is stored in pressurized CO_2_ cans provided by InnovaPrep.) The extraction fluid was collected, diluted with PBS, pH 7.4, for a total volume up to 12.3 mL, and assayed.

#### PEG/NaCl Precipitation

To the 100-mL spiked primary concentrate, 14 g PEG 8000 (Acros Organics, Geel, Belgium) and 1.17 g NaCl (for a final sample concentration of 0.2 M NaCl) were added and shaken vigorously by hand (~ 5 min, room temperature) until dissolved. Samples were then shaken overnight (16–18 h, 200 RPM, 4 °C), centrifuged (30 min, 4500×*g*, 4 °C), and the supernatant carefully removed. The pellets were resuspended in 10–20 mL PBS, pH 7.4 and assayed.

#### Skimmed-Milk Flocculation

Concentration by skimmed-milk flocculation was adapted from Calgua et al. 2008. Per 100-mL spiked primary concentrate, 1-mL 1% w/v skimmed-milk solution (Oxoid, Ltd., Hants, UK) was added. Samples were then pH adjusted to 3.0–4.0, shaken (4 h, 200 RPM, room temperature), centrifuged (30 min, 4500×*g*, 4 °C), and the supernatant carefully removed. The pellets were resuspended in 10–20 mL PBS, pH 7.4 and assayed.

### Skimmed-Milk Flocculation Investigations

#### Baseline Optimized Samples

The skimmed-milk flocculation method was optimized by evaluating different shaking times (4 h and overnight [16–18 h]) and temperatures (4 °C and room temperature [20–25 °C]). The samples were processed by addition of 1-mL 1% w/v skimmed-milk solution, followed by pH adjustment to 3.0–4.0. Samples were shaken, centrifuged (30 min, 4500×*g*, 4 °C), and the supernatant carefully removed. The pellets were resuspended in 10–20 mL PBS, pH 7.4, and assayed. No purification was conducted on these initial optimization samples.

Baseline optimized skimmed-milk flocculation samples included modifications from the preliminary method. These samples had an increased skimmed-milk concentration (5% w/v) and reduced shaking time (2 h) at room temperature. The baseline optimized skimmed-milk flocculation was performed by adding 1 mL 5% w/v skimmed-milk solution to 100-mL spiked primary concentrate, followed by pH adjustment to 3.0–4.0. Samples were shaken (2 h, 200 RPM, room temperature), centrifuged (30 min, 4500×*g*, 4 °C), and the supernatant was carefully removed. The pellets were resuspended in 10–20 mL PBS, pH 7.4, purified, and assayed. Purification was introduced on these baseline optimized samples.

#### Supplementary Investigations

Further experiments were performed to the baseline optimized skimmed-milk flocculation samples to explore effects upon conductivity and centrifuge speed adjustments. For conductivity investigations, the 100-mL primary concentrate was amended by adding 3.33 g artificial sea salts (Sigma-Aldrich, St. Louis, MO, USA) prior to spiking. For centrifuge method investigations, samples were centrifuged at 3500, 4000, and 4500×*g*.

#### PV1 De-aggregation

To address the greater than 100% recovery in some skimmed-milk flocculation samples, PV1 de-aggregation prior to seeding was investigated. De-aggregation was completed by filtering a 10^−2^ dilution (10^5^ PFU) of PV1 stock (10^7^ PFU) successively through a series of three syringe filters (0.45 µm polyethersulfone [PES], 0.22 µm PES, and 0.10 µm polyvinylidene fluoride) pre-wet with 0.01% Tween 20 (BDH, Merck, Darmstadt, Germany) in PBS, pH 7.4. Subsequent PV1 dilutions were made, and three PV1 levels (10^4^, 10^2^, and 10^1^ PFU) were spiked into primary concentrates. The primary concentrate was prepared as described previously, using 6-L volumes of influent wastewater. Experiments followed the baseline optimized skimmed-milk flocculation method previously described, with the exception of a reduced centrifuge speed (3500×*g*).

### Competitive Method Comparisons

The previously described PEG/NaCl precipitation method and the baseline optimized skimmed-milk flocculation (skimmed-milk concentration: 5% w/v; shaking conditions: 2 h at room temperature; centrifugation speed: 4500×*g*) method were compared for PV1 recovery from wastewater concentrate. Spiked primary concentrate samples were prepared as described above, mixed together, and then split into 100-mL volumes for secondary concentration. The two secondary concentration methods were performed on these seeded samples, followed by purification and enumeration.

### Purification

For the preliminary experiments and initial skimmed-milk flocculation optimization samples, no purification was performed prior to sample assay, and samples with cytotoxicity in more than half of assayed wells were not included in the analyses. Presence of cytotoxity in some samples during the preliminary investigation led to purification by Vertrel XF extraction for the subsequent skimmed-milk flocculation investigations and competitive method comparison (skimmed-milk and PEG/NaCl precipitation samples). For these samples, 1 mL Vertrel XF (E. I. du Pont de Nemours and Company, Wilmington, DE, USA) was added per 10 mL sample. Samples were vortexed (5 min), placed on ice (3 min), vortexed (5 min), and centrifuged (15 min, 3000×*g*, 4 °C). The aqueous phase was recovered, centrifuged (15 min, 3000×*g*, 4 °C), recovered, and assayed.

### Virus Plaque Assay and Enumeration

Viruses were enumerated by plaque assay on 95% confluent BGMK cell monolayers using 1.5% Avicel (FMC Health and Nutrition, Philadelphia, PA, USA) as the overlay media (Matrosovich et al. 2006). All assays were performed in duplicate or triplicate, with 200 µL of relevant dilutions in PBS, pH 7.4 plated onto 9.5 cm^2^ wells. Infected cells were incubated (40–48 h, 5% CO_2_, 37 °C) and stained (2% crystal violet in 20% methanol). Plaques were counted for infectious virus enumeration. PV1 percent recovery was determined by dividing the PV1 recovered by the PV1 seeded.

### Controls

Poliovirus was assumed to be absent from the primary concentrate as wild poliovirus transmission in the USA has not been observed since 1979 and most children under the age of five receive the inactivated polio vaccine (Strebel et al. 1992). However, as BGMK cells are not selective for poliovirus, negative controls of unseeded primary concentrate and relevant experimental reagents (e.g., PBS, beef extract/glycine eluent) were plated onto BGMK cells to confirm the absence of culturable non-polio enteroviruses (NPEV). Additionally, the PV1 seeding level (10^3^–10^4^ PFU/100 mL primary concentrate) was chosen to be at least an order of magnitude above the ambient NPEV concentrations detected in Seattle influent wastewater during this study period (not detected to 10^2^ PFU/100 mL primary concentrate) to minimize interference of NPEVs during assay. NPEVs were undetectable in all negative controls corresponding with the secondary concentration preliminary investigations, skimmed-milk flocculation investigations, and competitive method comparisons. A dilution series of the PV1 stock used for seeding was assayed as a positive control and to determine the seeded titer.

### Statistical Analysis

Statistical analyses were conducted using RStudio version 1.0.143 and Microsoft Excel 2016, and *p* values of < 0.05 were considered significant.

The one-way analysis of variance (ANOVA) test was used to determine whether there was a difference in virus recovery between the secondary concentration methods tested when three or more groups were compared. Tukey’s honest significant difference (HSD) test was used for post hoc evaluation of which group pairings were different.

Unpaired Student’s *t* tests were used to compare recoveries between competitive methods.

## Results

### Preliminary Investigations

**Fig. 1:**
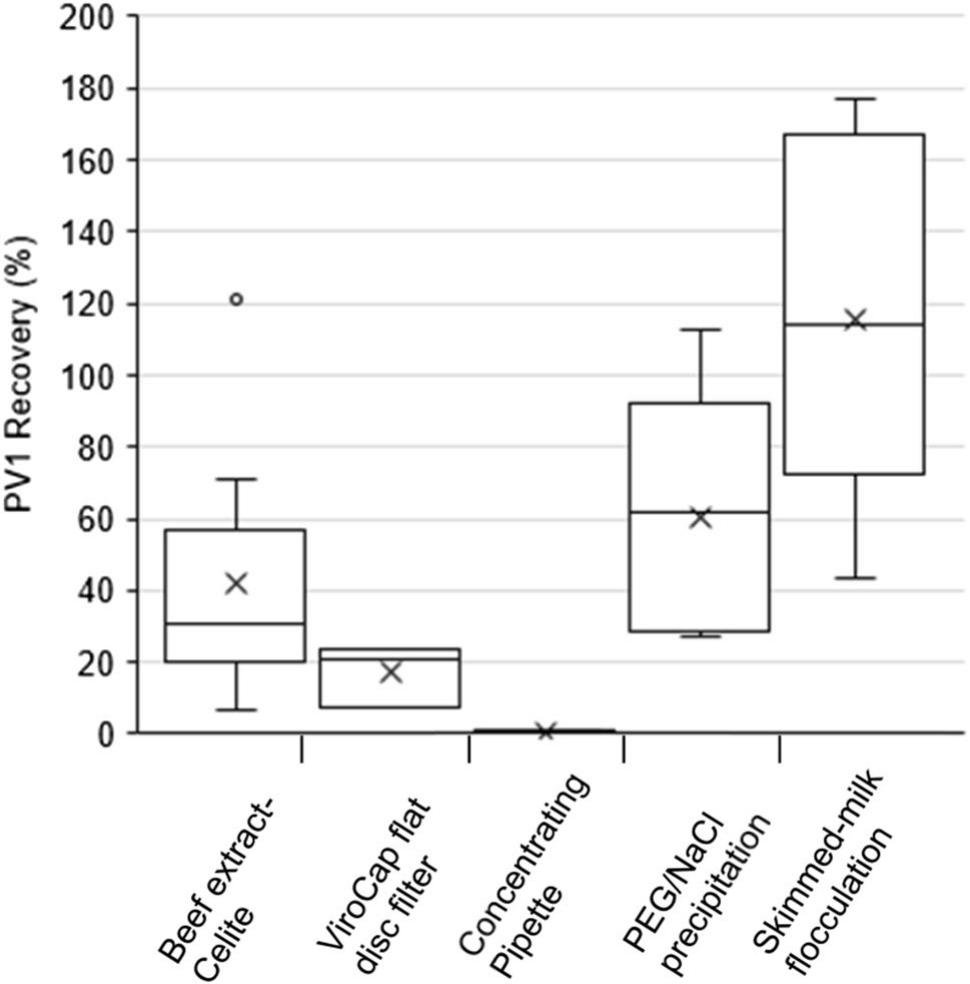
Preliminary secondary concentration PV1 percent recoveries from wastewater primary concentrates. Beef extract-Celite (*n* = 10); ViroCap flat disc filter (*n* = 3); concentrating pipette (*n* = 4); PEG/NaCl precipitation (*n* = 5); skimmed-milk flocculation (4 h shaking at room temperature) (*n* = 6). Box-and-whisker plot: lower and upper box lines show the first and third quartiles, respectively; middle box lines show the median; whiskers show the minimum and maximum; ‘×’ markers show the mean; circles show the outliers. *PV1* poliovirus type 1, *PEG* polyethylene glycol, *NaCl* sodium chloride

The mean PV1 recovery was higher when using PEG/NaCl precipitation (60.6%; *n* = 5) or skimmed-milk flocculation (116%; *n* = 6) than when using beef extract-Celite (41.8%; *n* = 10), ViroCap disc filter (17.2%; with average recovery of 222 ± 176% in the filtrate; *n* = 3), or Concentrating Pipette (0.32%; *n* = 4) (Fig. 1; Online Resource 2). Skimmed-milk flocculation resulted in significantly greater PV1 recovery than beef extract-Celite, ViroCap disc filter, and Concentrating Pipette (*p* = 0.003, 0.004, and < 0.001, respectively, ANOVA with Tukey’s HSD test). PEG/NaCl precipitation did not result in significantly different PV1 recovery when compared to beef extract-Celite, ViroCap flat disc filter, Concentrating Pipette, or skimmed-milk flocculation (*p* > 0.05, ANOVA with Tukey’s HSD test). Additional tests with the Concentrating Pipette indicated that there was no significant difference between the sample matrices and elution buffers used (*p* > 0.05, ANOVA with Tukey HSD test) (Online Resource 2).

Cytotoxicity was observed in 20.0%, 71.4%, and 18.8% of beef extract-Celite, PEG/NaCl precipitation, and skimmed-milk flocculation samples, respectively. Two of the PEG/NaCl precipitation samples (28.6%) were excluded from analyses, as cytotoxicity was observed in over half the assayed wells. For all other samples, cytotoxicity was observed in fewer than half the assayed wells and so the samples were included in the analyses. No cytotoxicity was observed in the ViroCap flat disc filter and Concentrating Pipette samples.

### Skimmed-Milk Flocculation Investigations

Initial optimization of the skimmed-milk flocculation method compared shaking temperatures and times (Online Resources 2 and 3). Overnight shaking at room temperature (52.0 ± 10.5%; *n* = 7) and at 4 °C (67.9 ± 43.2%; *n* = 3) resulted in similar PV1 recovery (*p* = 0.135, ANOVA with Tukey’s HSD test). Next, shaking at room temperature for 4 h resulted in a higher PV1 recovery (116 ± 52.7%; *n* = 6) when compared to shaking overnight (*p* = 0.010, ANOVA with Tukey HSD test).

Based on preliminary investigations and initial optimization of the skimmed-milk flocculation method, a short shaking time (2 h) at room temperature was used for the baseline optimized skimmed-milk flocculation samples. Additionally, a higher concentration of skimmed-milk solution was used (5% w/v). Beginning with these experiments, Vertrel purification was conducted. This resulted in PV1 recovery of 106 ± 24.8% (*n* = 8).

Further testing with sea salt amendments yielded a PV1 percent recovery of 70.9 ± 18.1% (*n* = 8), which was significantly lower than the PV1 recovery with no sea salts added (*p* = 0.017, *t* test). Centrifugation at 3500×*g*, 4000×*g*, and 4500×*g* did not result in significantly different PV1 recoveries (*p* > 0.05, ANOVA with Tukey HSD test; Table 1).

**Table 1:** Optimized skimmed-milk flocculation centrifuge speed analysis

Centrifuge speed (×*g*)	PV1 recovery (%)	
	Experiment 1	Experiment 2
3500	116	126
4000	88.7	109
4500	109	72.4

*PV1* poliovirus type 1

The addition of PV1 de-aggregation prior to seeding baseline optimized skimmed-milk flocculation samples was evaluated at three PV1 seeding levels. The mean PV1 percent recoveries for 10^4^, 10^2^, and 10^1^ PFU were 102 ± 29.7%, 114 ± 19.9%, and 107 ± 68.2%, respectively. These were not significantly different from baseline optimized skimmed-milk experiments with no de-aggregation (*p* > 0.05, ANOVA with Tukey HSD test).

### Competitive Method Comparison

**Fig. 2:**
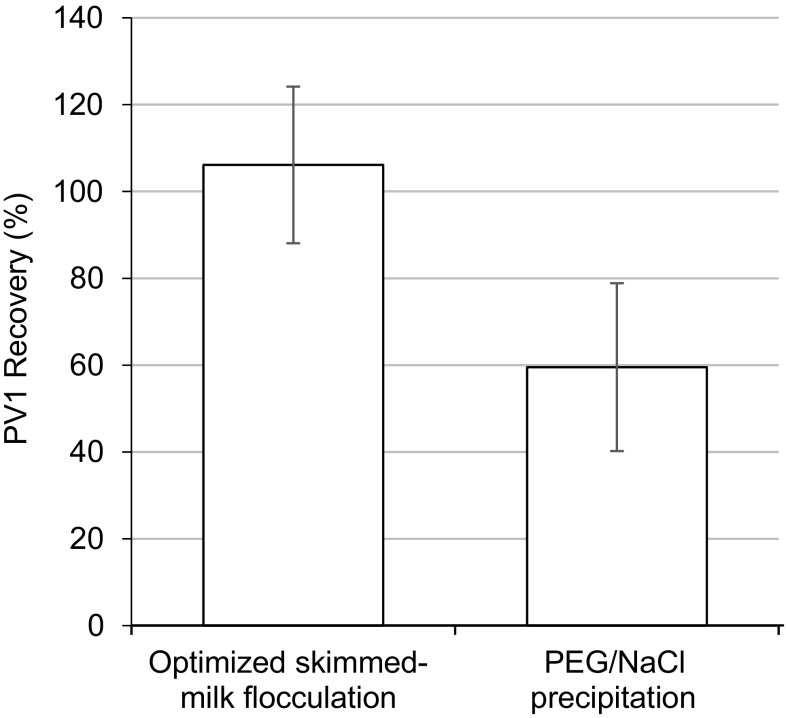
PV1 recovery from competitive secondary concentration methods: optimized skimmed-milk flocculation (*n* = 8) and PEG/NaCl precipitation (*n* = 8). Error bars represent 95% confidence intervals. *PV1* poliovirus type 1, *PEG* polyethylene glycol, *NaCl* sodium chloride. Optimized skimmed-milk flocculation includes 5% w/v skimmed-milk solution, 2 h shaking at room temperature (20–25 °C), and centrifugation at 4500×*g*

PEG/NaCl precipitation and skimmed-milk flocculation methods were compared in the competitive investigations. PV1 recovery by baseline optimized skimmed-milk flocculation (106 ± 24.8%, *n* = 8) was significantly higher than by PEG/NaCl precipitation (59.5 ± 19.6%, *n* = 8) (*p* = 0.004, *t* test; Fig. 2). Additionally, the processing requirements for these methods were compared (Table 2). Skimmed-milk flocculation requires low-cost reagents, standard laboratory equipment, and a relatively short processing time when compared with PEG/NaCl precipitation. It also enables shaking at room temperature, versus shaking at 4 °C for PEG/NaCl precipitation.

**Table 2:** Method comparison between PEG/NaCl precipitation and optimized skimmed-milk flocculation methods

Method	Reagent cost per 100 mL sample	Shaking time	Shaking temperature	Centrifuge speed
Optimized skimmed-milk flocculation	< $0.01 USD	2 h	Room temperature (20–25 °C)	3500–4500×*g*
PEG/NaCl precipitation	$1.66 USD	Overnight (16–18 h)	4 °C	4500×*g*

*PEG* polyethylene glycol, *NaCl* sodium chloride, *USD* United States dollar; Costs based on January 2018 prices in the USA

## Discussion

### Preliminary Investigations

In this study, several secondary concentration methods were identified and evaluated for their potential to improve poliovirus recovery and detection in wastewater. In the preliminary investigations, the method feasibility and initial viral recovery efficiencies were explored. The results determined that the beef extract-Celite, ViroCap flat disc filter, and Concentrating Pipette methods are non-competitive, as each resulted in low PV1 recoveries. However, the PEG/NaCl precipitation and skimmed-milk flocculation methods had statistically similar recoveries (*p* = 0.097, ANOVA with Tukey HSD test) and were therefore investigated in greater detail. Negative controls of the unspiked primary concentrate indicated that positive detection was due to spiked PV1 presence.

Although the beef extract-Celite method is simple to perform because it does not require refrigeration or centrifugation and requires only basic laboratory equipment (i.e., vacuum, graduated cylinder, membrane filter funnel), the recovery results were relatively low. Previous studies demonstrated that Celite (diatomaceous silica) can be used as a binding agent for poliovirus (Dahling and Wright 1986a, 1988; Rhodes et al. 2011). However, Dahling and Wright (1986a) reported significant variation in virus recovery based on Celite concentrations and beef extract lots (74–92% and 12–143%, respectively). Additionally, the study by Rhodes et al. (2011) tested the Celite concentration method by spiking poliovirus into tap water, unlike this study which used influent wastewater concentrate as a sample matrix. Organic matter in the wastewater and use of different reagent lots may have affected PV1-Celite binding, potentially contributing to the lower mean PV1 recovery obtained in this study (41.8%) relative to the Rhodes et al. (2011) (89.5%).

The ViroCap flat disc filter method was developed as a secondary concentration method during this study to further concentrate PV1 using the VIRADEL method. The low PV1 recovery obtained was likely attributed to challenges in filter capture (rather than elution recovery), which inherently limits the ViroCap disc filter method. The high PV1 recovery in the filtrate (222%) could be due to lack of PV1 capture on the filter, de-aggregation of PV1 virions during filtration, and/or the precision of the viral plaque assay. PV1 filter pass through is likely due to the composition of the feed water (diluted ViroCap primary concentrate: 0.25% beef extract, 8.3 mM glycine, pH 7.0–7.5). A beef extract/glycine solution is used for ViroCap filter elution, as the proteins block filter binding sites to release viruses from the filter media. Beef extract and glycine in the feed water may result in premature binding of filter sites on the ViroCap flat disc filter, thus inhibiting virus capture (Ikner et al. 2012). The greater than 100% PV1 recovery in the filtrate is likely due to de-aggregation of clumps of PV1 virions during filtration (Galasso and Sharp 1962; Fattal et al. 1977). This greater than 100% PV1 recovery could also be due to the inherent variability and lack of precision of viral plaque assay methods. The ViroCap flat disc filter samples did not result in cytotoxicity in viral plaque assays, which may be due to capture of organic inhibitors on the filters. This capture of organic matter may have also resulted in poor virus binding to the filter.

The Concentrating Pipette was investigated due to the method’s relative simplicity through automated and rapid processing. However, the instrument yielded a low PV1 recovery, which may be due to the capture of organic matter on the ultrafiltration tips. Additionally, the ultrafiltration pipette tip was only able to concentrate 50 mL of primary concentrate due to the filter tip fouling; therefore, two tips were required per sample. The use of a second pipette tip increased the surface area for potential virus losses, the sample dilution factor (thereby reducing the effective volume assayed), and the per-sample cost of materials to over 50 USD. Fagnant et al. (2017) demonstrated that double elution of 2″ ViroCap filters improved PV1 recovery, while resulting in a total primary concentrate volume of 200 mL. Concentrating this higher volume (200 mL) using the Concentrating Pipette method would significantly affect the method efficiency, effective volume assayed, and cost. These concerns, combined with a high initial investment of ~ 10,000 USD, demonstrated that the Concentrating Pipette method did not warrant further evaluation for this application.

The PEG/NaCl precipitation method was conducted as described by Fagnant et al. (2018) with the exception of a reduced centrifuge speed from (6000×*g* to 4500×*g*) to accommodate for laboratories with lower centrifuge speed capacity. This method was not further optimized, as it has been well characterized for enteric viruses in complex water matrices (Yamamoto et al. 1970; Minor 1985; Schwab et al. 1995; van Zyl et al. 2006). Changes to the PEG and NaCl concentrations, shaking time, and shaking temperature were not evaluated. Further investigations exploring these variables may further improve PV1 recovery using the PEG/NaCl precipitation method.

The preliminary skimmed-milk flocculation method was adapted from Calgua et al. (2008) and simplified for easier adoption by laboratories with limited resources. The total flocculation time was reduced from 16 h (8 h shaking + 8 h sedimentation) to 4 h (shaking only), and the centrifuge speed was reduced from 7000×*g* to 4500×*g*.

High PV1 recoveries for the PEG/NaCl precipitation and skimmed-milk flocculation samples demonstrated the potential of these methods for poliovirus recovery from wastewater samples. However, these results were impacted by cytotoxicity observed in the viral plaque assay. To compensate for the cytotoxicity, a higher dilution factor was plated (1:100 vs. 1:10), resulting in a reduced effective volume assayed and sensitivity. Purification was incorporated into later investigations to reduce cytotoxicity. However, this added purification step means results from the preliminary and competitive investigations are not directly comparable.

### Skimmed-Milk Flocculation Investigations

The skimmed-milk flocculation method was improved by examining (1) the concentration of the skimmed-milk solution added, (2) shaking time, (3) addition of artificial sea salts, and (4) centrifuge speed. First, the skimmed-milk powder solution was increased to 5% (w/v) and, second, the shaking time decreased to 2-h at room temperature. As sample acidification facilitates floc formation and PV1 capture, increasing the skimmed-milk concentration increased the number of available flocs to which PV1 could bind. The mean PV1 recoveries are statistically similar when using the preliminary method (1% skimmed-milk with 4-h shaking) (116%, *n* = 6) and the baseline optimized method (5% skimmed-milk with 2-h shaking) (106%, *n* = 8; *p* = 0.666, *t* test). Future research could consider comparing the two concentrations with a 2-h shaking time. Third, the addition of artificial sea salts was evaluated in skimmed-milk flocculation samples, as water samples with conductivity levels ≤ 1.5 mS cm^−1^ have been found to negatively affect the adsorption of viral particles to flocs (Calgua et al. 2013). While influent wastewater conductivity can range from 0.5 to 1.5 mS cm^−1^, the beef extract/glycine eluate may have increased the conductivity of the primary concentrate to greater than 1.5 mS cm^−1^ although the conductivity of the primary concentrates was not measured (Levlin and Hultman 2008). In this study, the addition of artificial sea salts resulted in a lower PV1 recovery. Future investigation should evaluate the effect of the sea salt composition and the conductivity of different sample matrices on poliovirus recovery. Fourth, the baseline optimized skimmed-milk flocculation process was further investigated by varying the centrifuge speed to explore the usability in laboratory settings where centrifuge speeds are limited. Results suggest that reducing the centrifuge speed to 3500 x *g*does not impact PV1 recovery. Future investigations should consider evaluating viral recovery at further lowered speeds.

De-aggregation of the seeded PV1 stock was also applied to the baseline optimized skimmed-milk flocculation samples to investigate virus aggregation effects upon the greater than 100% recovery from these samples. The de-aggregated samples did not significantly impact PV1 recovery when compared to samples that were not de-aggregated. This indicates that either de-aggregation was not the primary factor contributing to the greater than 100% recovery or the de-aggregation step was insufficient to break up virus aggregates. This high recovery could also be due to the inherent variability and lack of precision of viral plaque assay methods. Negative controls suggest that the presence of other viruses in the sample matrix did not contribute to the high PV1 recovery.

### Competitive Methods

Evaluation of the PEG/NaCl precipitation and baseline optimized skimmed-milk flocculation (skimmed-milk concentration: 5% w/v; shaking conditions: 2 h at room temperature; centrifugation speed: 4500×*g*) samples indicated that the skimmed-milk flocculation samples resulted in higher PV1 recovery. Introduction of a purification step in the competitive experiments reduced observed cytotoxicity in all sample types. However, purification did not significantly impact PV1 recovery on PEG/NaCl precipitation samples, compared to the preliminary investigations (*p* = 0.944, *t* test).

Of the methods examined, the baseline optimized skimmed-milk flocculation method is recommended for secondary concentration of poliovirus from ViroCap filter eluates because it offers the greatest recovery of PV1 while using a relatively simple protocol. The method requires low-cost reagents, standard laboratory equipment, shaking at room temperature, and a relatively short processing time. Additionally, the organic flocculation pH adjustments were completed using inexpensive, disposable pH strips, further demonstrating the method’s inherent simplicity. These advantages may be particularly important in low-resource laboratory settings.

### Limitations

Several limitations existed in this experimental design, including (1) the wastewater matrix used, (2) possible viral aggregation, (3) the ability of other non-polio viruses to amplify on the cell line used, and (4) use of a single test organism. These concerns were addressed to the best means possible. First, the wastewater composition may have varied slightly across preliminary experimental samples, as water was collected at varying times of day and across different seasons (Wang et al. 2017). This matrix variation may have affected the PV1 recovery from these samples (Fagnant et al. 2014, 2017). To control for this variation, the same composite primary concentrate was used for direct comparisons during the competitive method experiments (PEG/NaCl precipitation and optimized skimmed-milk flocculation). Additionally, as the wastewater was collected from the same location throughout the study, it is not anticipated that the variation would be substantial enough to result in selection of a different competitive method. Second, viral aggregation may have contributed to a greater than 100% PV1 recovery (Galasso and Sharp 1962; Fattal et al. 1977, 2017; Teunis et al. 2005). While pre-filtration of the PV1 stock prior to spiking attempted to reduce aggregation effects, the recovery was still greater than 100%. The greater than 100% recovery could also be due to limitations in recovery quantification precision from virus plaque assays. Future investigations could determine the extent of virus aggregation by coupling culture-based assays with molecular or microscopy methods (Gerba and Betancourt 2017). Third, the viral plaque assay used has inherent limitations, as the BGMK cell line was optimized for enterovirus assays and is not selective for poliovirus (Dahling and Wright 1986b). Therefore, it is possible that NPEVs were present in the influent wastewater and detected in the plaque assay. To account for this concern, each assay included unseeded primary concentrate as a negative control and experiments were seeded with PV1 at least an order of magnitude above ambient NPEV concentrations in Seattle influent wastewater during this study period. NPEVs were undetectable in the negative controls corresponding with the secondary concentration preliminary investigations, skimmed-milk flocculation investigations, and competitive method comparisons, indicating that NPEVs did not impact the PV1 recovery. Finally, while PV1 was used as a surrogate for enteric viruses in this work, the virus size, shape, proteins, and charge interactions may impact recovery of a given organism. Thus, the PV1 recovery reported in this study may not be directly applicable to other target organisms. Skimmed-milk flocculation has been evaluated for concentrating a variety of viruses, bacteria, and protozoa from water sources (Gonzales-Gustavson et al. 2017), and recovery of these organisms from the unique primary concentrate matrix should be investigated further. These limitations, combined with a lower number of sample replicates in the preliminary investigations, indicate further investigations while controlling more variables (e.g., a homogenous composite primary concentrate used across all samples) may be valuable.

## Conclusions

This exploration into secondary concentration of PV1 from ViroCap filter primary concentrate demonstrated that skimmed-milk flocculation yields a high PV1 recovery while maintaining a relatively inexpensive and simple protocol. Future work should evaluate secondary concentration methods for recovery of other viruses from primary concentrate (e.g., poliovirus types 2 and 3, rotavirus, norovirus, and hepatitis A virus) and examine the applicability of these methods for recovery of bacteria (e.g., *S. enterica* serotype Typhi and *Vibrio cholerae*), and protozoa (e.g., *Giardia lamblia* and *Cryptosporidium parvum*). Field-testing is also recommended to compare skimmed-milk flocculation with PEG/NaCl precipitation as a secondary concentration step for BMFS samples.

## Electronic supplementary material

Below is the link to the electronic supplementary material.

Concentrating Pipette optimization methods, results and discussion. Skimmed-milk flocculation optimization results and discussion. (DOCX 19 KB)

Table S1. Poliovirus type 1 (PV1) recovery for secondary concentration preliminary investigations. (DOCX 21 KB)

Fig. S1 Poliovirus type 1 (PV1) recovery from preliminary skimmed-milk flocculation by shaking time and temperature. (DOCX 17 KB)
