# Supplementary material for: Evaluation of Secondary Concentration Methods for Poliovirus Detection in Wastewater
Source: Food Environ Virol. 2019 Jan 5;11(1):20–31. doi: 10.1007/s12560-018-09364-y (PMC6394643; doi:10.1007/s12560-018-09364-y)
Supplement: Supplementary file 1 — Concentrating Pipette optimization methods, results and discussion. Skimmed-milk flocculation optimization results and discussion. (DOCX 19 KB) [file 12560_2018_9364_MOESM1_ESM.docx]

ELECTRONIC SUPPLEMENTARY MATERIAL: ONLINE RESOURCE 1

*Evaluation of secondary concentration methods for poliovirus detection in wastewater*

Jill C. Falman^1^, Christine S. Fagnant-Sperati^1^, Alexandra L. Kossik^1^, David S. Boyle^2^, John Scott Meschke^1^*

^1^ Department of Environmental & Occupational Health Sciences, University of Washington, 4225 Roosevelt Way NE, Suite 100, Seattle, WA 98195 USA

^2^ PATH, 2201 Westlake Avenue, Suite 200, Seattle, WA 98121 USA

*Corresponding Author: J. Scott Meschke, jmeschke@uw.edu; phone: +1-206-221-5470

*Submitted to Food and Environmental Virology*

**Table of Contents**

[**Methods** 3](#_Toc523393566)

[**Results and Discussion** 3](#_Toc523393567)

# **Methods**

*Concentrating Pipette*

In addition to the spiked primary concentrate, concentration by the Concentrating Pipette (InnovaPrep, Drexel, MO, USA) was performed on fresh 1.5% beef extract, 0.05 M glycine, pH 7.0 solution and on PBS, pH 7.4. These proxy matrices were examined due to challenges in filtering the primary concentrate. The 100-mL spiked matrix (beef extract/glycine solution or PBS) was divided into two 50-mL aliquots for concentration, as all these matrices resulted in fouling of the hollow fiber polysulfone pipette tips after processing 50 mL. Samples were concentrated with the following custom instrument settings: valve open, 35; valve closed, 100; pulse count, 2; flow buffer, 12; and extraction delay, 6. The beef extract/glycine matrix was extracted with six to ten extractions using PBS, pH 9.5 (*n*=5), or Dulbecco’s Modified Eagle’s Medium (DMEM) (*n*=1). The PBS matrix was extracted with one to two extractions using 0.075% Tween 20 Tris (HC08001, InnovaPrep) (*n*=4). Each extraction used approximately 1 to 1.5 mL of extraction fluid (fluid is stored in pressurized CO_2_ cans provided by InnovaPrep). The extraction fluid was collected, additionally diluted with PBS for a total volume up to 12.3 mL with PBS, pH 7.4, and assayed.

# **Results and Discussion**

*Concentrating Pipette*

The average PV1 recovery from the PBS matrix was 2.97 ± 2.38% (*n*=4). In contrast, the PV1 recovery from the fresh beef extract/glycine solution samples was higher, with recoveries of 61.5 ± 79.5% (*n*=5) using Tween/PBS, pH 9.5 extraction fluid and 24.4% (*n*=1) using DMEM extraction fluid.

Multiple extractions were conducted and different extraction fluids were used in an attempt to improve PV1 recovery, as poliovirus recovery has not been previously characterized by the Concentrating Pipette. Five to nine additional extractions were performed on samples with the beef extract/glycine matrix, compared to samples with the PBS and primary concentrate matrices. Although PV1 recovery was highest among these samples that used more extractions, the “clean” matrix and different elution fluids may have also contributed to the higher recovery values. The use of Tween/PBS pH 9.5 as an extraction fluid with the beef extract/glycine matrix yielded the highest recovery, however, the matrix effects of wastewater in the primary concentrate should be determined with this extraction method. The Concentrating Pipette was not further evaluated due to the per-sample high cost and personnel time requirements.

*Preliminary skimmed-milk flocculation optimization*

Initial optimization of the skimmed-milk flocculation method was investigated, as poliovirus recovery from complex water matrices has not been previously characterized by this relatively new method (Online Resource 3 Fig. S1). These experiments demonstrated that shaking for 4 hours at room temperature resulted in the highest PV1 recovery. The lower PV1 recovery after overnight shaking, compared to 4-hour shaking, could be due to loss of virus viability over time. As the samples were concentrated from influent wastewater, it is possible presence of bacteria or fungi in the sample negatively impacted virus viability. Activity of other organisms would be suppressed at cooler temperatures (4°C), which may explain why the overnight samples at room temperature resulted in the lowest PV1 recovery. It is also possible that room temperature enhanced the flocculation process, resulting in increased virus adsorption to the flocs. This may explain the improved performance of the method shaking overnight at room temperature compared to 4°C.
