# Supplementary material for: Evaluation of Secondary Concentration Methods for Poliovirus Detection in Wastewater
Source: Food Environ Virol. 2019 Jan 5;11(1):20–31. doi: 10.1007/s12560-018-09364-y (PMC6394643; doi:10.1007/s12560-018-09364-y)
Supplement: Supplementary file 2 — Table S1. Poliovirus type 1 (PV1) recovery for secondary concentration preliminary investigations. (DOCX 21 KB) [file 12560_2018_9364_MOESM2_ESM.docx]

ELECTRONIC SUPPLEMENTARY MATERIAL: ONLINE RESOURCE 2

*Evaluation of secondary concentration methods for poliovirus detection in wastewater*

Jill C. Falman^1^, Christine S. Fagnant-Sperati^1^, Alexandra L. Kossik^1^, David S. Boyle^2^, John Scott Meschke^1^*

^1^ Department of Environmental & Occupational Health Sciences, University of Washington, 4225 Roosevelt Way NE, Suite 100, Seattle, WA 98195 USA

^2^ PATH, 2201 Westlake Avenue, Suite 200, Seattle, WA 98121 USA

*Corresponding Author: J. Scott Meschke, jmeschke@uw.edu; phone: +1-206-221-5470

*Submitted to Food and Environmental Virology*

**Table S1.** PV1 recovery for secondary concentration preliminary investigations

| Method | Sample Matrix | *n* | Elution fluid | Shaking time and temperature | Wastewater sample collection date (dd/mm/yy) | PV1 recovery (%) | Mean PV1 recovery  (%) |
| --- | --- | --- | --- | --- | --- | --- | --- |
| Beef-extract-Celite | Primary concentrate | 10 | PBS, pH 9.0 | N/A | 01/03/16 | 19.3 | 41.8 |
|  |  |  |  |  | 02/04/16 | 52.3 |  |
|  |  |  |  |  | 02/04/16 | 121 |  |
|  |  |  |  |  | 27/05/16 | 6.50 |  |
|  |  |  |  |  | 14/06/16 | 20.0 |  |
|  |  |  |  |  | 14/06/16 | 30.5 |  |
|  |  |  |  |  | 14/06/16 | 71.1 |  |
|  |  |  |  |  | 16/06/16 | 30.4 |  |
|  |  |  |  |  | 04/08/16 | 26.7 |  |
|  |  |  |  |  | 04/08/16 | 39.6 |  |
| ViroCap flat disc filter | Primary concentrate | 3 | 1.5% beef extract, 0.05 M glycine, pH 9.5 | N/A | 05/01/16 | 7.23 | 17.2 |
|  |  |  |  |  | 05/01/16 | 20.8 |  |
|  |  |  |  |  | 05/01/16 | 23.7 |  |
| Concentrating Pipette | Primary concentrate | 4 | Tween/Tris | N/A | 31/08/15 | 0.00 | 0.32 |
|  |  |  |  |  | 31/08/15 | 0.07 |  |
|  |  |  |  |  | 31/08/15 | 0.44 |  |
|  |  |  |  |  | 31/08/15 | 0.77 |  |
|  | 1.5% beef extract, 0.05 M glycine, pH 7.0 | 5 | PBS pH 9.5 | N/A | N/A | 14.3 | 61.5 |
|  |  |  |  |  |  | 21.4 |  |
|  |  |  |  |  |  | 24.2 |  |
|  |  |  |  |  |  | 83.0 |  |
|  |  |  |  |  |  | 165 |  |
|  |  | 1 | DMEM | N/A | N/A | 24.4 | 24.4 |
|  | PBS, pH 7.4 | 4 | Tween/Tris | N/A | N/A | 1.64 | 24.7 |
|  |  |  |  |  |  | 1.77 |  |
|  |  |  |  |  |  | 3.88 |  |
|  |  |  |  |  |  | 4.60 |  |
| Skimmed-milk flocculation | Primary concentrate | 6 | N/A | 4 hours,  room temperature | 01/03/16 | 43.5 | 116 |
|  |  |  |  |  | 20/06/16 | 122 |  |
|  |  |  |  |  | 20/06/16 | 177 |  |
|  |  |  |  |  | 24/11/16 | 82.4 |  |
|  |  |  |  |  | 24/11/16 | 106 |  |
|  |  |  |  |  | 24/11/16 | 164 |  |
|  |  | 7 | N/A | Overnight,  room temperature | 01/03/16 | 58.2 | 52.0 |
|  |  |  |  |  | 11/03/16 | 37.2 |  |
|  |  |  |  |  | 11/03/16 | 51.3 |  |
|  |  |  |  |  | 31/03/16 | 63.3 |  |
|  |  |  |  |  | 01/06/16 | 64.6 |  |
|  |  |  |  |  | 03/06/16 | 52.3 |  |
|  |  |  |  |  | 20/06/16 | 36.9 |  |
|  |  | 3 | N/A | Overnight,  4ºC | 31/03/16 | 51.5 | 67.9 |
|  |  |  |  |  | 01/06/16 | 86.2 |  |
|  |  |  |  |  | 03/06/16 | 66.0 |  |
| PEG/NaCl precipitation | Primary concentrate | 5 | N/A | Overnight,  4ºC | 01/03/16 | 30.1 | 60.6 |
|  |  |  |  |  | 11/03/16 | 62.1 |  |
|  |  |  |  |  | 11/03/16 | 113 |  |
|  |  |  |  |  | 03/06/16 | 27.2 |  |
|  |  |  |  |  | 20/06/16 | 71.0 |  |

PV1, poliovirus type 1; PBS, phosphate-buffered saline; N/A, not applicable; DMEM, Dulbecco’s Modified Eagle Medium; room temperature, 20-25°C; overnight, 16-18 hours; PEG, polyethylene glycol; NaCl, sodium chloride.
