# Supplementary material for: Evaluation of Secondary Concentration Methods for Poliovirus Detection in Wastewater
Source: Food Environ Virol. 2019 Jan 5;11(1):20–31. doi: 10.1007/s12560-018-09364-y (PMC6394643; doi:10.1007/s12560-018-09364-y)
Supplement: Supplementary file 3 — Fig. S1 Poliovirus type 1 (PV1) recovery from preliminary skimmed-milk flocculation by shaking time and temperature. (DOCX 17 KB) [file 12560_2018_9364_MOESM3_ESM.docx]

ELECTRONIC SUPPLEMENTARY MATERIAL: ONLINE RESOURCE 3

*Evaluation of secondary concentration methods for poliovirus detection in wastewater*

Jill C. Falman^1^, Christine S. Fagnant-Sperati^1^, Alexandra L. Kossik^1^, David S. Boyle^2^, John Scott Meschke^1^*

^1^ Department of Environmental & Occupational Health Sciences, University of Washington, 4225 Roosevelt Way NE, Suite 100, Seattle, WA 98195 USA

^2^ PATH, 2201 Westlake Avenue, Suite 200, Seattle, WA 98121 USA

*Corresponding Author: J. Scott Meschke, jmeschke@uw.edu; phone: +1-206-221-5470

*Submitted to Food and Environmental Virology*

**Fig. S1** Poliovirus type 1 (PV1) recovery from preliminary skimmed-milk flocculation by shaking time and temperature. Error bars represent 95% confidence intervals. 4 hours, RT (n=6); overnight, RT (n=7); overnight, 4°C (n=3); overnight, 16-18 hours; RT, room temperature (20-25°C)
